# The Interaction Between HDL-C Level and HNF4A rs4812829 on Incident Type 2 Diabetes Risk in a Chinese Cohort

**DOI:** 10.3390/nu18142270

**Published:** 2026-07-11

**Authors:** Haodong Zhang, Yixin Li, Yinxi Tan, Huangda Guo, Hexiang Peng, Yi Zheng, Yiqun Wu, Xueying Qin, Tao Wu, Dafang Chen, Yonghua Hu, Mengying Wang

**Affiliations:** 1 Department of Nutrition and Food Hygiene, School of Public Health, Peking University, Beijing 100191, China; 2010306222@stu.pku.edu.cn (H.Z.); 2511110213@pku.edu.cn (Y.L.); tanyinxi@bjmu.edu.cn (Y.T.); 1910306230@pku.edu.cn (Y.Z.); 2 Department of Epidemiology and Health Statistics, School of Public Health, Peking University, Beijing 100191, China; 2411110182@pku.edu.cn (H.G.); qywu118@163.com (Y.W.); xueyingqin@bjmu.edu.cn (X.Q.); twu@bjmu.edu.cn (T.W.); dafangchen@bjmu.edu.cn (D.C.); yhhu@bjmu.edu.cn (Y.H.); 3 Key Laboratory of Epidemiology of Major Diseases, Ministry of Education, Peking University, Beijing 100191, China; 4 Department of Epidemiology and Health Statistics, Xiangya School of Public Health, Central South University, Changsha 410012, China; 225125@csu.edu.cn

**Keywords:** *HNF4A*, T2D, HDL-C, gene–lipid interaction, rural Chinese population

## Abstract

**Background/Objectives**: To evaluate the association between *HNF4A* rs4812829 and type 2 diabetes (T2D) in a rural Chinese population and to investigate its interaction with blood lipids in the association. **Methods**: A total of 4496 participants free of diabetes at baseline from a family-based cohort in rural China were included. Demographic, lifestyle, and medical history data were collected via standardized questionnaires. Anthropometric and biochemical measurements were performed using standardized protocols and automated assays on fasting blood samples. Mixed-effects Cox proportional hazards models, accounting for familial clustering, were employed to examine the association between *HNF4A* rs4812829 and incident T2D risk. Additionally, multiplicative interaction terms were used to assess interactions. **Results**: After a median follow-up of 10.76 years, 895 incident T2D cases were identified. Under an additive genetic model, each additional G allele of rs4812829 was significantly associated with an increased risk of T2D (HR 1.38, 95% CI 1.19–1.59). A significant multiplicative interaction was observed between rs4812829 and HDL-C (*p* = 0.004). In genotype-stratified analyses, higher HDL-C levels were strongly associated with lower T2D risk among AA homozygotes (HR 0.04, 95% CI 0.003–0.43) and AG heterozygotes (HR 0.30, 95% CI 0.10–0.84), but not among GG homozygotes (HR 1.32, 95% CI 0.35–4.91). **Conclusions**: This study finds *HNF4A* intronic variant rs4812829 is significantly associated with the incident T2D risk in a rural Chinese population, and this association exhibits an interaction with HDL-C levels. These findings support further investigation of *HNF4A*-related lipid pathways and require replication in independent Chinese and multi-ancestry cohorts before genetic and lipid profiles can be considered for T2D risk stratification.

## 1. Introduction

Over the past three decades, the global burden of diabetes has continually increased. As of 2021, approximately 529 million people worldwide were affected by diabetes, with type 2 diabetes (T2D) accounting for 96.0% of these cases [1]. Similarly, diabetes prevalence in China has risen markedly in recent years. A nationwide survey conducted between 2018 and 2019 involving 173,642 participants from 31 provinces in mainland China reported a diabetes prevalence of 12.4%, of which over 90% was T2D [2].

T2D is a complex, multifactorial disease influenced by a combination of lifestyle, environmental, and genetic factors. Although obesity and lifestyle-related factors such as physical inactivity and unhealthy dietary habits significantly contribute to T2D risk, genetic predisposition also plays a critical role in its pathogenesis [3]. Genome-wide association studies (GWAS) have identified more than 600 genetic loci associated with T2D susceptibility [4]. Among these loci, hepatocyte nuclear factor 4 alpha (*HNF4A*) has attracted considerable attention. *HNF4A* is involved in regulating glucose metabolism, lipid metabolism, and protein synthesis in the liver, as well as in modulating insulin and other hormone synthesis and secretion in the pancreas [5,6,7]. Previous investigations have reported associations of mutations in *HNF4A* with increased risk of maturity-onset diabetes of the young (MODY) and T2D [5,8]. Notably, the intronic single nucleotide polymorphism (SNP) rs4812829 in *HNF4A* reached genome-wide significance for T2D in South Asian discovery cohorts and was replicated in a Saudi Arabian population, with supportive evidence also reported in Pakistani and Indian cohorts across T2D-related phenotypes [9,10,11,12].

*HNF4A* is a key regulator of lipid metabolism, being required for normal triglyceride (TG) and cholesterol homeostasis [13]. Consistent with this biology, dyslipidemia, including hypercholesterolemia, hypertriglyceridemia, and low high-density lipoprotein cholesterol (HDL-C), is associated with a higher risk of incident T2D than normolipidemia [14,15,16]. Clinically and mechanistically, higher TG and low-density lipoprotein cholesterol (LDL-C) concentrations correlate with higher blood glucose, and excess cholesterol can induce apoptosis of pancreatic β cells via oxidative stress, linking lipid imbalance to diabetogenesis [17,18].

Although substantial research has explored the association between *HNF4A* rs4812829 and T2D, related studies specifically focusing on the Chinese population are comparatively limited. Additionally, whether rs4812829 interacted with lipids in the association with T2D has been less explored. To address this gap, the present study draws on a large family-based cohort in rural northern China to estimate the association between rs4812829 and incident T2D. We further examined the interactions between rs4812829 and individual blood lipid components (LDL-C, HDL-C, and TG) on T2D risk. Clarifying interactions between rs4812829 and lipid levels in rural Chinese populations may refine etiological understanding and provide a basis for future studies on risk stratification in genetically susceptible individuals.

## 2. Materials and Methods

### 2.1. Study Participants

This analysis used data from an ongoing family-based cohort in Fangshan District (southwestern Beijing), where families were the sampling units. Baseline assessments were conducted from June 2005 to August 2017, with two follow-ups (July 2019–December 2020 and December 2023–July 2024). The master protocol has been described elsewhere [19]. This study has been approved by the Peking University Biomedical Ethics Committee (IRB00001052-13027). Individuals with extreme outliers in critical variables (dietary intake, anthropometry, biochemical markers, or age) or who were unable to participate because of severe comorbid conditions were excluded. After applying these criteria, 4496 participants were included.

### 2.2. Blood Sampling and Genotyping Quality Control

Participants underwent private interviews, standardized physical examinations, and biospecimen collection by trained medical staff. Venous blood was drawn for DNA extraction and genotyping. During variant-level quality control, SNPs with minor allele frequency < 1% or deviating from Hardy–Weinberg equilibrium (*p* < 1.0 × 10^−6^, evaluated among unrelated founders) were excluded [19]. For the lead variant analyzed in this study (*HNF4A* rs4812829), the minor allele was A with MAF = 0.3539, and in unrelated founders, HWE chi-square test yielded *p* = 0.02.

### 2.3. Definitions and Measurements

T2D was defined by self-report confirmed by study physicians or by abnormal glycaemic markers: fasting plasma glucose ≥ 7.0 mmol/L, HbA1c ≥ 6.5%, or use of glucose-lowering medication. Covariates, including age, sex, occupation, marital status, education, alcohol consumption, smoking, sedentary time, physical activity, sleep duration and timing, hypertension history, family history of diabetes, and cardiovascular disease history, were obtained using the standardized questionnaire administered within the FISSIC cohort protocol [19]. Dietary intake was assessed with a semi-quantitative food-frequency questionnaire (FFQ) developed and implemented within the FISSIC cohort [19], which captured consumption frequency and portion size. Height and weight were measured to the nearest 0.1 cm and 0.1 kg using a calibrated stadiometer–scale. Waist circumference was measured to the nearest 0.1 cm at 1 cm above the umbilicus with a non-stretchable tape. Biochemical measurements, including serum levels of HDL-C, TG, and LDL-C, were performed on fasting blood samples using standardized automated assays.

A composite healthy lifestyle score was constructed following our previous work [20]: never smoking, no alcohol consumption, healthy diet score above the cohort median, physical activity above the median, sedentary time below the median, 7–8 h of sleep, and morning chronotype. Each favorable factor contributed one point (range 0–7); score categories were ≤2 (low, 0), 3–5 (medium, 1), and 6–7 (high, 2) [20].

### 2.4. Statistical Analysis

Analyses were conducted using R software version 4.4.2 (R Foundation for Statistical Computing, Vienna, Austria), with the coxme package for mixed-effects Cox models. Given within-family correlation, single-variable differences by T2D status were tested with marginal generalized estimating equations (family code as the cluster ID; exchangeable working correlation; sandwich standard errors): Gaussian models for continuous variables (log-transformed if skewed) and binomial models for binary variables; results are reported as mean ± SD, median (IQR), or counts (percentages).

Genotypes at *HNF4A* rs4812829 were coded under three inheritance models. Additive: AA, AG, GG coded 0, 1, 2 (G-allele dosage, the GWAS effect allele, whereas the minor allele in our cohort was A). Dominant: AG + GG = 1 vs. AA = 0. Recessive: GG = 1 vs. AA + AG = 0.

Associations with incident T2D were estimated using mixed-effects Cox models with a family-level random intercept to account for intrafamilial clustering (package coxme). Person-time accrued from baseline to the earliest time of T2D diagnosis, loss to follow-up, death, or study end. We fitted three hierarchical models: Model 1 adjusted for age and sex; Model 2 additionally included the healthy lifestyle score, log-transformed household income, and BMI (kg/m^2^); Model 3 further adjusted for baseline hypertension, cardiovascular diseases, log-transformed LDL-C, TG, HDL-C, and parental history of diabetes. Effect sizes are reported as hazard ratios (HRs) with 95% confidence intervals (CIs).

To assess effect modification by blood lipid components, multiplicative product terms (lipid component × rs4812829 genotype) were included in Model 3, with statistical significance evaluated by Wald tests. Three prespecified lipid traits (LDL-C, HDL-C, and TG) were tested as candidate effect modifiers under the additive genetic model; we therefore applied a Bonferroni correction for these three interaction tests, with a corrected significance threshold of α = 0.05/3 = 0.0167. A two-sided α = 0.05 denoted nominal significance. The proportional-hazards assumption was evaluated by fitting marginal coxph models with family-clustered robust standard errors and testing scaled Schoenfeld residuals. For sensitivity analyses, we repeated the models after excluding baseline users of lipid-lowering therapy to address potential treatment-related confounding of lipid levels.

## 3. Results

### 3.1. Baseline Characteristics

The mean age of the participants was 56.97 ± 10.14 years, and 51.5 percent were men. During follow-up, 895 individuals developed T2D, yielding a cumulative incidence of 19.9%. Table 1 compares baseline characteristics by incident T2D status. Participants who developed T2D were slightly older than those who remained free of diabetes, although the difference was not statistically significant (*p* = 0.39). They exhibited a less favorable adiposity profile, with higher mean BMI and waist circumference (*p* < 0.001). A higher proportion of participants with incident T2D were non-farmers (*p* = 0.001) and married (*p* = 0.03). Individuals who later developed T2D had a higher prevalence of hypertension (*p* < 0.001) and prior cardiovascular diseases (*p* < 0.001). They also had higher TG (*p* = 0.002) and lower HDL-C (*p* < 0.001). TC was slightly lower (*p* = 0.02). Smoking status, alcohol intake, composite health score, family history of diabetes, LDL-C, and educational attainment did not differ significantly between groups (all *p* > 0.05).

### 3.2. Association Between HNF4A rs4812829 and Incident Type 2 Diabetes

The *HNF4A* rs4812829 variant was significantly associated with an increased risk of incident T2D. In the primary additive model, each additional copy of the G-allele conferred a 38% higher risk of T2D in the fully adjusted analysis (HR 1.38, 95% CI 1.19–1.59; *p* < 0.001). A clear dose-response relationship was observed in the genotype-specific analysis. Compared to individuals with the AA genotype, the fully adjusted HRs were 1.36 (95% CI 1.16–1.59) for AG heterozygotes and 1.89 (95% CI 1.41–2.53) for GG homozygotes (both *p* < 0.001).

The findings were consistent in other genetic models. In the fully adjusted dominant model, carrying at least one G-allele was associated with a higher risk (HR 1.36, 95% CI 1.03–1.80; *p* = 0.03). In the recessive model, GG homozygotes showed a significantly increased risk compared to carriers of the A-allele (HR 1.61, 95% CI 1.31–1.96; *p* < 0.001). Results were robust across all levels of adjustment. The associations between *HNF4A* rs4812829 genotypes and incident T2D under additive, dominant, and recessive genetic models are shown in Table 2.

### 3.3. Interaction of HDL-C and HNF4A rs4812829 Genotype on Incident Type 2 Diabetes

In mixed-effects Cox models specifying a multiplicative interaction between rs4812829 (additive dosage) and base-10 log–transformed HDL-C, the interaction was statistically significant in the fully adjusted model (*p* for interaction = 0.004), indicating that the inverse association between HDL-C and T2D risk weakens as the G-allele count increases. The rs4812829 × HDL-C interaction remained statistically significant after Bonferroni correction for the three prespecified lipid interaction tests (nominal *p* = 0.004; Bonferroni-adjusted *p* = 0.012), whereas interactions with LDL-C (*p* = 0.26) and TG (*p* = 0.95) were not significant before or after correction (Supplementary Table S5).

**Figure 1:**
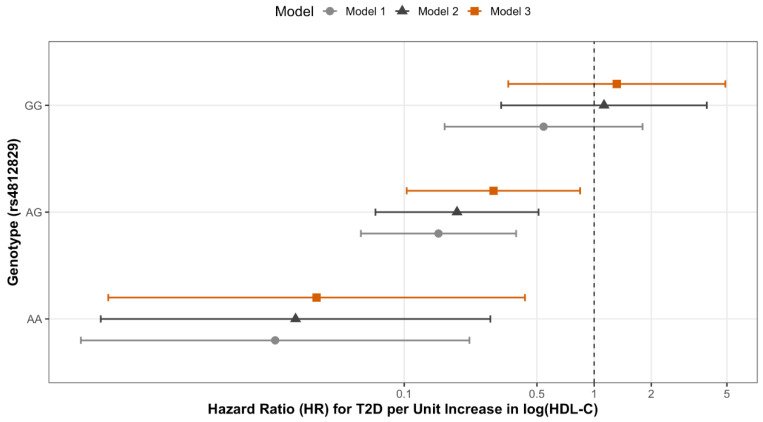
HDL-C Effect on Type 2 diabetes Risk Stratified by *HNF4A* rs4812829 Genotype.

Genotype-stratified analyses were directionally consistent. Among AA homozygotes, higher HDL-C was associated with lower risk across sequential adjustments (Model 3 HR = 0.04, 95% CI 0.003–0.43, *p* = 0.01). This large estimate reflects the log10 HDL-C scale and should be interpreted cautiously; after rescaling HDL-C per 1-SD, the AA estimate was more moderate (HR = 0.61, 95% CI 0.42–0.88; Supplementary Table S6), with genotype-specific sample sizes and event counts shown in Supplementary Table S3. A significant inverse association was also observed in AG heterozygotes (Model 3 HR = 0.30, 95% CI 0.10–0.84, *p* = 0.02). No significant association was observed among GG homozygotes in any model (Model 3 HR = 1.32, 95% CI 0.35–4.91, *p* = 0.68). These genotype-stratified associations are illustrated in Figure 1. By contrast, interactions of rs4812829 with LDL-C and with TG were not statistically significant in any specification (all *p* for interaction > 0.05).

### 3.4. Sensitivity Analyses

To address potential treatment-related confounding of lipid levels, we further repeated the analyses after excluding baseline users of lipid-lowering therapy. The main finding remained robust. Under additive coding, the per-G-allele association remained significant across adjustments. The recessive model also remained significant, whereas the dominant model was not statistically significant after full adjustment (Supplementary Table S1). The genotype-stratified associations of HDL-C with T2D mirrored the primary pattern—strongly inverse in AA, attenuated in AG, and null in GG (Supplementary Figure S1).

## 4. Discussion

Using data from this prospective, family-based cohort study in rural northern China, we found that rs4812829 showed a dose–response association with T2D under additive and recessive encodings. We further identified a significant multiplicative interaction between rs4812829 and HDL-C, whereby the protective association of higher HDL-C with lower T2D risk was progressively attenuated with increasing G-allele dosage. Genotype-stratified estimates were directionally consistent with this interaction.

Parental diabetes history showed a non-significant but directionally higher T2D incidence (21.0% vs 19.6%); this does not conflict with the rs4812829 association because family history is a coarse proxy affected by underdiagnosis, recall error, and shared environmental risk. Farmers had lower crude T2D incidence and slightly higher HDL-C than non-farmers, potentially reflecting occupational activity and correlated lifestyle or socioeconomic factors; although occupation adjustment did not materially change the HDL-C interaction, residual confounding remains possible. Our observed dose-response association between rs4812829 and incident T2D is consistent with prior evidence implicating *HNF4A* in β-cell dysfunction and diabetes risk, and extends this evidence to a rural Chinese population. This signal, tagged by intronic variants such as rs4812829, had attributes consistent with impaired β-cell function [12]. Signals at or near *HNF4A* have also been reported in Middle Eastern cohorts [21], with supportive signals across South Asian subpopulations and related phenotypes [10,22]. Beyond common variation, rare-variant analyses in >420,000 individuals demonstrate that pathogenic *HNF4A* alleles (notably p.Arg114Trp) substantially elevate T2D risk and map *HNF4A* onto the spectrum from monogenic (MODY1) to polygenic diabetes [23].

Two complementary pathways support a role for *HNF4A* in glycaemic regulation. First, the β-cell pathway indicates that *HNF4A* participates in a β-cell transcriptional network that includes cross-regulation with *HNF1A* and governs glucose-stimulated insulin secretion (GSIS). Rare monogenic defects and islet perturbation experiments indicate that reduced *HNF4A* activity impairs insulin gene expression and the insulin secretory machinery, which provides a direct route to hyperglycaemia [5,12,23]. Second, the hepatic lipid pathway shows that *HNF4A* acts as a master regulator of TG and cholesterol homeostasis, influencing VLDL export and cholesterol efflux programs such as *ABCA1* and *APOA1*. Liver-specific Hnf4α knockdown in mice lowers plasma TG, TC, and HDL, with down-regulation of Abca1 and Apoa1 and defective VLDL secretion, while human hepatocyte studies show that *HNF4A* directly up-regulates *ABCA1* under cholesterol depletion [13,24]. Consistently, carriers of rare *HNF4A* risk alleles exhibit a coordinated adverse cardiometabolic profile characterized by higher glucose, TG, and LDL-C and lower apoA and HDL-C [23], supporting a liver-centric mechanism through which *HNF4A* variation can influence glycaemic risk.

Against this backdrop, the observed genotype and HDL-C interaction in our data fits a genetic–lipid coupling model. Upstream positioning of *HNF4A* over hepatocellular cholesterol efflux and lipoprotein biogenesis provides a transcriptional basis for between-genotype differences in the availability and functionality of HDL particles [13,24]. In parallel, HDL and its apolipoproteins augment β-cell function and survival via *ABCA1*/*ABCG1*/SR-BI–dependent lipid homeostasis signalling, enhancing insulin mRNA and GSIS and exerting cytoprotective effects [25,26]. When *HNF4A* activity is reduced or when risk alleles are present, the β-cell benefits of higher HDL may be blunted, resulting in the graded attenuation observed in our data from AA to AG to GG. Population data in older adults showing that low HDL states amplify the impact of T2D susceptibility alleles provide convergent epidemiological support for such modification [27]. Findings in Pima Indians, where *CETP*, *HNF4A*, and *KLF14* variants showed sex dependent associations with T2D and several signals remained after adjustment for HDL-C, are consistent with partial modification rather than full mediation through HDL pathways. This pattern aligns with the genotype-dependent attenuation observed in our study [28]. Because genotype precedes adult lipid levels, lipids were not treated as classical confounders but as potential downstream traits and effect modifiers; lipid-adjusted models therefore estimate lipid-profile-independent associations rather than mediation effects. Nevertheless, we caution that the causal regulatory targets of rs4812829 remain to be identified. Future work should integrate cis-eQTL and colocalization analyses across liver and islet, as well as single-cell multi-omics and perturbational assays, to resolve the underlying mechanisms.

Strengths include the prospective, family-based design with explicit modeling of intrafamilial clustering, long follow-up, and rigorous hierarchical adjustment with sensitivity analyses. By contributing data from a rural Chinese cohort and quantifying a genotype and HDL-C interaction at *HNF4A*, this study helps fill an evidence gap and may inform future studies integrating genetic susceptibility with metabolic profiling.

This study has several limitations. First, as an observational analysis, residual confounding and measurement error cannot be fully excluded. Second, rs4812829 is located within a linkage disequilibrium block, and the possibility that its observed effects reflect correlated variants or long-range regulatory influences cannot be ruled out. Third, rs4812829 departed from HWE equilibrium in our cohort. This may partly reflect that participants were recruited from the same rural area using family-based sampling, which can introduce relatedness and local population structure into genotype distributions. Although our mixed-effects models accounted for familial clustering and the findings were directionally consistent in sensitivity analyses, genotyping error or residual population structure cannot be fully excluded; therefore, the genetic findings should be interpreted cautiously and replicated in independent populations.

## 5. Conclusions

In conclusion, this prospective family-based cohort supports an association between *HNF4A* rs4812829 and incident T2D and suggests a potential interaction with HDL-C. These results motivate further functional investigation of *HNF4A*-related pathways and suggest that combining genetic and lipid profiles may enhance T2D risk stratification; however, replication in independent Chinese and multi-ancestry cohorts and functional validation are needed before such approaches can be translated to precision prevention.

## Figures and Tables

**Table 1:** Baseline Characteristics of Participants by Incident Type 2 diabetes Status.

Baseline Characteristics	Total (*n* = 4496)	T2D (*n* = 895)	Non-T2D (*n* = 3601)	*p*-Value
Age, years, mean (SD)	56.97 ± 10.14	57.22 ± 9.37	56.91 ± 10.32	0.39
Gender, *n* (%)				0.27
Male	2315 (51.5)	476 (53.2)	1839 (51.1)	
Female	2181 (48.5)	419 (46.8)	1762 (48.9)	
BMI (kg/m^2^), mean (SD)	25.77 ± 3.40	26.49 ± 3.28	25.59 ± 3.40	<0.001
Waist circumference (cm), mean (SD)	90.02 ± 9.93	92.59 ± 9.72	89.38 ± 9.88	<0.001
Occupation, *n* (%)				0.001
Farmer	1889 (42.0)	324 (36.2)	1565 (43.5)	
Non-Farmer	2574 (57.3)	568 (63.5)	2006 (55.7)	
Education level, *n* (%)				0.85
Junior high school and below	712 (15.8)	145 (16.2)	567 (15.7)	
High school and above	3784 (84.2)	750 (83.8)	3034 (84.3)	
Marital status, *n* (%)				0.03
Married	3914 (87.1)	802 (89.6)	3112 (86.4)	
Unmarried	550 (12.2)	91 (10.2)	459 (12.7)	
Smoke never, *n* (%)	2356 (52.4)	463 (51.7)	1893 (52.6)	0.70
Drink never, *n* (%)	2793 (62.1)	553 (61.8)	2240 (62.2)	0.77
Health score, mean (SD)	1.03 ± 0.45	1.03 ± 0.47	1.03 ± 0.44	0.86
Hypertension, *n* (%)	2987 (66.4)	647 (72.3)	2340 (65.0)	<0.001
Cardiovascular diseases, *n* (%)	2196 (48.8)	510 (57.0)	1686 (46.8)	<0.001
Family diabetes history (≥1 parent), *n* (%)	945 (21.0)	198 (22.1)	747 (20.7)	0.52
TC (mmol/L), median [25%,75%]	3.20 [2.64–3.84]	3.12 [2.55–3.65]	3.23 [2.67–3.90]	0.02
TG (mmol/L), median [25%,75%]	1.22 [0.81–1.68]	1.24 [0.85–1.75]	1.20 [0.80–1.67]	<0.01
HDL-C (mmol/L), median [25%,75%]	0.96 [0.78–1.21]	0.94 [0.75–1.11]	0.97 [0.79–1.23]	<0.001
LDL-C (mmol/L), median [25%,75%]	2.33 [1.81–2.87]	2.31 [1.78–2.81]	2.33 [1.82–2.89]	0.53

BMI, body mass index; TC, total cholesterol; TG, triglycerides; HDL-C, high-density lipoprotein cholesterol; LDL-C, low-density lipoprotein cholesterol.

**Table 2:** Association of *HNF4A* rs4812829 Genotypes with Incident Type 2 diabetes.

	Genotype	Model 1		Model 2		Model 3	
		HR (95% CI)	*p*-Value	HR (95% CI)	*p*-Value	HR (95% CI)	*p*-Value
Additive Model	AA	1.00		1.00		1.00	
	AG	1.36 (1.18–1.58)	<0.001	1.36 (1.18–1.57)	<0.001	1.38 (1.19–1.59)	<0.001
	GG	1.86 (1.40–2.48)	<0.001	1.85 (1.38–2.47)	<0.001	1.89 (1.41–2.53)	<0.001
Dominant Model	AA	1.00		1.00		1.000	
	AG + GG	1.34 (1.01–1.77)	0.04	1.361 (1.03–1.80)	0.03	1.36 (1.03–1.80)	0.03
Recessive Model	AA + AG	1.00		1.00		1.00	
	GG	1.60 (1.31–1.95)	<0.001	1.57 (1.29–1.92)	<0.001	1.61 (1.31–1.96)	<0.001

## Data Availability

The datasets generated and analyzed during the current study are not publicly available due to privacy and ethical restrictions involving genetic and health information of cohort participants, as well as the data-sharing terms specified in the informed consent and approved by the Peking University Biomedical Ethics Committee (IRB00001052-13027). De-identified data supporting the findings of this study are available from the corresponding author (M.W., mywang@bjmu.edu.cn) upon reasonable request and subject to approval by the cohort’s steering committee.

## Supplementary Materials

The following supporting information can be downloaded at: https://www.mdpi.com/article/10.3390/nu18142270/s1, Table S1: Sensitivity analysis of the association between *HNF4A* rs4812829 genotypes and incident type 2 diabetes after excluding baseline users of lipid-lowering therapy; Table S2: Summary of the analysis population and availability of *HNF4A* rs4812829 genotype data; Table S3: Genotype-specific sample size, incident type 2 diabetes events, person-years, and HDL-C distribution for *HNF4A* rs4812829; Table S4: Incident type 2 diabetes events across HDL-C tertiles stratified by *HNF4A* rs4812829 genotype; Table S5: Interaction analyses between *HNF4A* rs4812829 and lipid traits for incident type 2 diabetes risk with multiple-testing correction; Table S6: Genotype-stratified associations of HDL-C with incident type 2 diabetes risk on log10-transformed and per-SD scales; Table S7: Crude incidence of type 2 diabetes according to parental history of diabetes; Table S8: Occupation-specific distributions of incident type 2 diabetes, HDL-C, physical activity, and sedentary time; Figure S1: HDL-C effect on incident type 2 diabetes risk stratified by *HNF4A* rs4812829 genotype after excluding baseline users of lipid-lowering therapy; Figure S2: Schoenfeld PH Test.

## Abbreviations

The following abbreviations are used in this manuscript:

***ABCA1***	ATP-binding cassette transporter A1
***ABCG1***	ATP-binding cassette subfamily G member 1
***A******POA1***	Apolipoprotein A1
**BMI**	Body mass index
**CI**	Confidence interval
**DNA**	Deoxyribonucleic acid
**eQTL**	Expression quantitative trait locus
**FFQ**	Food-frequency questionnaire
**FISSIC**	Fangshan/Family-based Ischemic Stroke Study in China
**GSIS**	Glucose-stimulated insulin secretion
**GWAS**	Genome-wide association study
**HbA1c**	Glycated hemoglobin A1c
**HDL-C**	High-density lipoprotein cholesterol
***HNF1A***	Hepatocyte nuclear factor 1 alpha
***HNF4A***	Hepatocyte nuclear factor 4 alpha
**HR**	Hazard ratio
**HWE**	Hardy–Weinberg equilibrium
**IQR**	Interquartile range
**LDL-C**	Low-density lipoprotein cholesterol
**MAF**	Minor allele frequency
**MODY**	Maturity-onset diabetes of the young
**PH**	Proportional hazards
**SD**	Standard deviation
**SNP**	Single nucleotide polymorphism
**SR-BI**	Scavenger receptor class B type I
**T2D**	Type 2 diabetes
**TC**	Total cholesterol
**TG**	Triglyceride
**VLDL**	Very-low-density lipoprotein

## References

[1] GBD 2021 Diabetes Collaborators (2023). Global, regional, and national burden of diabetes from 1990 to 2021, with projections of prevalence to 2050: A systematic analysis for the Global Burden of Disease Study 2021. Lancet.

[2] Wang L., Peng W., Zhao Z., Zhang M., Shi Z., Song Z., Zhang X., Li C., Huang Z., Sun X. (2021). Prevalence and Treatment of Diabetes in China, 2013–2018. JAMA.

[3] Fuchsberger C., Flannick J., Teslovich T.M., Mahajan A., Agarwala V., Gaulton K.J., Ma C., Fontanillas P., Moutsianas L., McCarthy D.J. (2016). The genetic architecture of type 2 diabetes. Nature.

[4] Suzuki K., Hatzikotoulas K., Southam L., Taylor H.J., Yin X., Lorenz K.M., Mandla R., Huerta-Chagoya A., Melloni G.E.M., Kanoni S. (2024). Genetic drivers of heterogeneity in type 2 diabetes pathophysiology. Nature.

[5] Ng N.H.J., Ghosh S., Bok C.M., Ching C., Low B.S.J., Chen J.T., Lim E., Miserendino M.C., Tan Y.S., Hoon S. (2024). HNF4A and HNF1A exhibit tissue specific target gene regulation in pancreatic beta cells and hepatocytes. Nat. Commun..

[6] Wang H., Maechler P., Antinozzi P.A., Hagenfeldt K.A., Wollheim C.B. (2000). Hepatocyte nuclear factor 4alpha regulates the expression of pancreatic beta -cell genes implicated in glucose metabolism and nutrient-induced insulin secretion. J. Biol. Chem..

[7] Yeh M.M., Bosch D.E., Daoud S.S. (2019). Role of hepatocyte nuclear factor 4-alpha in gastrointestinal and liver diseases. World J. Gastroenterol..

[8] Mahajan A., Wessel J., Willems S.M., Zhao W., Robertson N.R., Chu A.Y., Gan W., Kitajima H., Taliun D., Rayner N.W. (2018). Refining the accuracy of validated target identification through coding variant fine-mapping in type 2 diabetes. Nat. Genet..

[9] Shabana, Ullah Shahid S., Wah Li K., Acharya J., Cooper J.A., Hasnain S., Humphries S.E. (2016). Effect of six type II diabetes susceptibility loci and an FTO variant on obesity in Pakistani subjects. Eur. J. Hum. Genet..

[10] Kanthimathi S., Chidambaram M., Bodhini D., Liju S., Bhavatharini A., Uma R., Anjana R.M., Mohan V., Radha V. (2017). Association of recently identified type 2 diabetes gene variants with Gestational Diabetes in Asian Indian population. Mol. Genet. Genom..

[11] Binjawhar D.N., Ansari M.G.A., Sabico S., Hussain S.D., Alenad A.M., Alokail M.S., Al-Masri A.A., Al-Daghri N.M. (2023). Genetic Variants of HNF4A, WFS1, DUSP9, FTO, and ZFAND6 Genes Are Associated with Prediabetes Susceptibility and Inflammatory Markers in the Saudi Arabian Population. Genes.

[12] Kooner J.S., Saleheen D., Sim X., Sehmi J., Zhang W., Frossard P., Been L.F., Chia K.S., Dimas A.S., Hassanali N. (2011). Genome-wide association study in individuals of South Asian ancestry identifies six new type 2 diabetes susceptibility loci. Nat. Genet..

[13] Yin L., Ma H., Ge X., Edwards P.A., Zhang Y. (2011). Hepatic hepatocyte nuclear factor 4α is essential for maintaining triglyceride and cholesterol homeostasis. Arter. Thromb. Vasc. Biol..

[14] Peng J., Zhao F., Yang X., Pan X., Xin J., Wu M., Peng Y.G. (2021). Association between dyslipidemia and risk of type 2 diabetes mellitus in middle-aged and older Chinese adults: A secondary analysis of a nationwide cohort. BMJ Open.

[15] Yan Z., Xu Y., Li K., Liu L. (2024). Association between high-density lipoprotein cholesterol and type 2 diabetes mellitus: Dual evidence from NHANES database and Mendelian randomization analysis. Front. Endocrinol..

[16] Cheng C., Liu Y., Sun X., Yin Z., Li H., Zhang M., Zhang D., Wang B., Ren Y., Zhao Y. (2019). Dose-response association between the triglycerides: High-density lipoprotein cholesterol ratio and type 2 diabetes mellitus risk: The rural Chinese cohort study and meta-analysis. J. Diabetes.

[17] Wang L., Yan N., Zhang M., Pan R., Dang Y., Niu Y. (2022). The association between blood glucose levels and lipids or lipid ratios in type 2 diabetes patients: A cross-sectional study. Front. Endocrinol..

[18] Lu X., Liu J., Hou F., Liu Z., Cao X., Seo H., Gao B. (2011). Cholesterol induces pancreatic β cell apoptosis through oxidative stress pathway. Cell Stress Chaperones.

[19] Tang X., Hu Y., Chen D., Zhan S., Zhang Z., Dou H. (2007). The Fangshan/Family-based Ischemic Stroke Study In China (FISSIC) protocol. BMC Med. Genet..

[20] Guo H., Peng H., Wang S., Hou T., Li Y., Zhang H., Jiang J., Ma B., Wang M., Wu Y. (2024). Healthy Lifestyles Modify the Association of Melatonin Receptor 1B Gene and Ischemic Stroke: A Family-Based Cohort Study in Northern China. J. Pineal Res..

[21] Al-Daghri N.M., Alkharfy K.M., Alokail M.S., Alenad A.M., Al-Attas O.S., Mohammed A.K., Sabico S., Albagha O.M. (2014). Assessing the contribution of 38 genetic loci to the risk of type 2 diabetes in the Saudi Arabian Population. Clin. Endocrinol..

[22] Jan A., Zakiullah, Ali S., Muhammad B., Arshad A., Shah Y., Bahadur H., Khan H., Khuda F., Akbar R. (2023). Decoding type 2 diabetes mellitus genetic risk variants in Pakistani Pashtun ethnic population using the nascent whole exome sequencing and MassARRAY genotyping: A case-control association study. PLoS ONE.

[23] Huerta-Chagoya A., Schroeder P., Mandla R., Li J., Morris L., Vora M., Alkanaq A., Nagy D., Szczerbinski L., Madsen J.G.S. (2024). Rare variant analyses in 51,256 type 2 diabetes cases and 370,487 controls reveal the pathogenicity spectrum of monogenic diabetes genes. Nat. Genet..

[24] Ohoka N., Okuhira K., Cui H., Wu W., Sato R., Naito M., Nishimaki-Mogami T. (2012). HNF4α increases liver-specific human ATP-binding cassette transporter A1 expression and cholesterol efflux to apolipoprotein A-I in response to cholesterol depletion. Arter. Thromb. Vasc. Biol..

[25] Fryirs M.A., Barter P.J., Appavoo M., Tuch B.E., Tabet F., Heather A.K., Rye K.A. (2010). Effects of high-density lipoproteins on pancreatic beta-cell insulin secretion. Arter. Thromb. Vasc. Biol..

[26] von Eckardstein A., Widmann C. (2014). High-density lipoprotein, beta cells, and diabetes. Cardiovasc. Res..

[27] Sullivan S.O., Al Hageh C., Henschel A., Chacar S., Abchee A., Zalloua P., Nader M. (2024). HDL levels modulate the impact of type 2 diabetes susceptibility alleles in older adults. Lipids Health Dis..

[28] Nair A.K., Piaggi P., McLean N.A., Kaur M., Kobes S., Knowler W.C., Bogardus C., Hanson R.L., Baier L.J. (2016). Assessment of established HDL-C loci for association with HDL-C levels and type 2 diabetes in Pima Indians. Diabetologia.

