# Supplementary material for: The Interaction Between HDL-C Level and HNF4A rs4812829 on Incident Type 2 Diabetes Risk in a Chinese Cohort
Source: Nutrients. 2026 Jul 11;18(14):2270. doi: 10.3390/nu18142270 (PMC13414583; doi:10.3390/nu18142270)
Supplement: Supplementary file 1 [file nutrients-18-02270-s001.zip › nutrients-4390748-supplementary.pdf]

Supplementary Table S1. Sensitivity Analysis: Association of *HNF4A* rs4812829 Genotypes With Incident T2DM After Excluding Baseline Users of Lipid-lowering Therapy

	Genotype	HR (95% CI), Model 1	P-value	HR (95% CI), Model 2	P-value	HR (95% CI), Model 3	P-value
Additive Model	AA	1.00		1.00		1.00	
	AG	1.33(1.14-1.55)	<0.001	1.32(1.13-1.54)	<0.001	1.35(1.15-1.57)	<0.001
	GG	1.76(1.30-2.39)	<0.001	1.75(1.29-2.37)	<0.001	1.81(1.33-2.46)	<0.001
Dominant Model	AA	1.00		1.00		1.00	
	AG+GG	1.21(0.91-1.62)	0.19	1.22(0.91-1.63)	0.18	1.23(0.92-1.65)	0.16
Recessive Model	AA+AG	1.00		1.00		1.00	
	GG	1.60(1.29-1.97)	<0.001	1.58(1.28-1.96)	<0.001	1.63(1.31-2.02)	<0.001

Model specification: Mixed-effects Cox proportional-hazards models with a family-level random intercept (coxme). Model 1 adjusted for age and sex; Model 2 additionally included healthy-lifestyle score, log household income, and BMI; Model 3 further included baseline hypertension, CVD, TG, HDL-C, LDL-C, and parental history of diabetes. Hazard ratios (HRs) with 95% confidence intervals (CIs) are reported.

Supplementary Table S2. Analysis dataset summary

Total(n)	T2d_events(n)	enotyped_rs4812829(n)
4,496	895	2,546

Supplementary Table S3. Genotype-specific sample size and incident T2D events for *HNF4A* rs4812829

Genotype	Total(n)	Incident_T2D(n)	non_T2D (n)	Crude_incidence (%)	Incidence_rate_per_1000	HDLC_median	Lipid_lowering_users (n)
AA	390	68	322	17.44	14.78	1.03	20
AG	1,022	176	846	17.22	14.43	1.03	69
GG	1,134	231	903	20.37	20.35	1.06	99

Supplementary Table S4. Incident T2D events by rs4812829 genotype and HDL-C tertile

Genotype	HDLC_tertile	Total (n)	Incident_T2D (n)	Non_T2D (n)	Crude_incidence (%)	HDLC_min	HDLC_max
AA	T1_low	124	31	93	25.00	0.39	0.94
AA	T2_mid	143	24	119	16.78	0.94	1.19
AA	T3_high	123	13	110	10.57	1.20	1.90
AG	T1_low	338	75	263	22.19	0.03	0.94
AG	T2_mid	340	62	278	18.24	0.94	1.20
AG	T3_high	344	39	305	11.34	1.20	1.90
GG	T1_low	387	83	304	21.45	0.37	0.94
GG	T2_mid	366	85	281	23.22	0.94	1.20
GG	T3_high	381	63	318	16.54	1.20	1.90

Supplementary Table S5. Primary lipid interaction tests with Bonferroni correction

Subset	Lipid_trait	HR (95% CI)	P-value	*P*_Bonferroni	*P*_FDR
Full cohort	HDL-C	5.93 (1.76-19.96)	0.004	0.01	0.01
	LDL-C	1.88 (0.63-5.59)	0.26	0.77	0.39
	TG	0.98 (0.52-1.85)	0.95	1.00	0.95
Excluding baseline lipid-lowering therapy users	HDL-C	6.03 (1.69-21.53)	0.006	0.02	0.02
	LDL-C	2.48 (0.75-8.27)	0.14	0.42	0.21
	TG	1.11 (0.57-2.16)	0.77	1.00	0.77
Full cohort (adjusted for occupation)	HDL-C	5.75 (1.70-19.42)	0.005	0.01	0.01
	LDL-C	1.98 (0.67-5.89)	0.22	0.65	0.33
	TG	0.95 (0.50-1.78)	0.86	1.00	0.86

Supplementary Table S6. Genotype-stratified HDL-C associations reported on per-SD scales

Subset	Model	Genotype	HR(95%CI)	P-value
Full cohort	Model 1	AA	0.56 (0.40-0.80)	0.001
	Model 1	AG	0.64 (0.53-0.77)	<0.001
	Model 1	GG	0.91 (0.77-1.07)	0.24
	Model 2	AA	0.58 (0.41-0.83)	0.002
	Model 2	AG	0.67 (0.56-0.81)	<0.001
	Model 2	GG	1.00 (0.85-1.18)	1.00
	Model 3	AA	0.61 (0.42-0.87)	0.01
	Model 3	AG	0.72 (0.59-0.87)	0.001
	Model 3	GG	1.02 (0.86-1.21)	0.82
Excluding lipid-lowering therapy users	Model 1	AA	0.56 (0.40-0.80)	0.002
	Model 1	AG	0.64 (0.53-0.78)	<0.001
	Model 1	GG	0.91 (0.77-1.08)	0.28
	Model 2	AA	0.59 (0.41-0.84)	0.003
	Model 2	AG	0.67 (0.55-0.82)	<0.001
	Model 2	GG	0.99 (0.84-1.18)	0.94
	Model 3	AA	0.63 (0.43-0.91)	0.01
	Model 3	AG	0.70 (0.57-0.86)	0.001
	Model 3	GG	1.01 (0.84-1.21)	0.92

Supplementary Table S7. Family history of diabetes summary

Family_DM	Total (n)	Incident_T2D (n)	Crude_incidence (%)	HDLC_median
>=1 parent	945	198	20.952	0.970
No parent	3,551	697	19.628	0.960

Supplementary Table S8. Occupation, physical activity proxies, sedentary time, and HDL-C

Occupation	Total(n)	Incident_T2D(n)	Crude_incidence (%)	HDL-C, median [Q1, Q3]	Sport total, median [Q1, Q3] hours/day	Sedentary time, median [Q1, Q3] hours/day
Farmer	1889	324	17.15	1.020 [0.870, 1.280]	0.000 [0.000, 0.800]	2.000 [1.500, 4.000]
Non-farmer	2574	568	22.07	0.930 [0.730, 1.140]	0.429 [0.000, 1.000]	3.000 [2.000, 5.000]

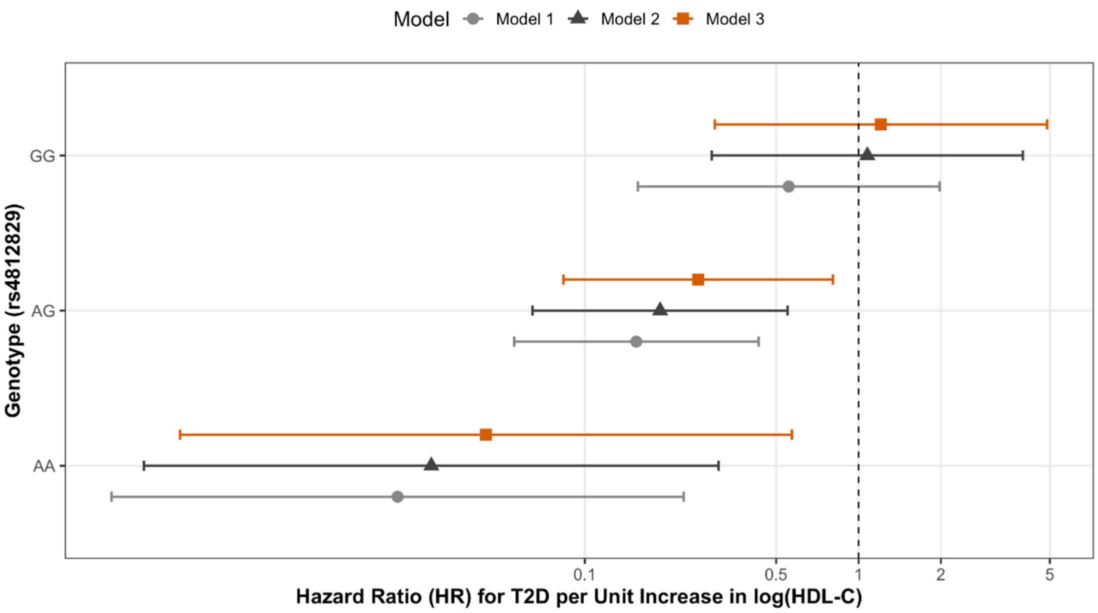

Supplementary Figure S1. HDL-C effect on T2DM risk stratified by *HNF4A* rs4812829 Genotype After Excluding Baseline Users of Lipid-lowering Therapy

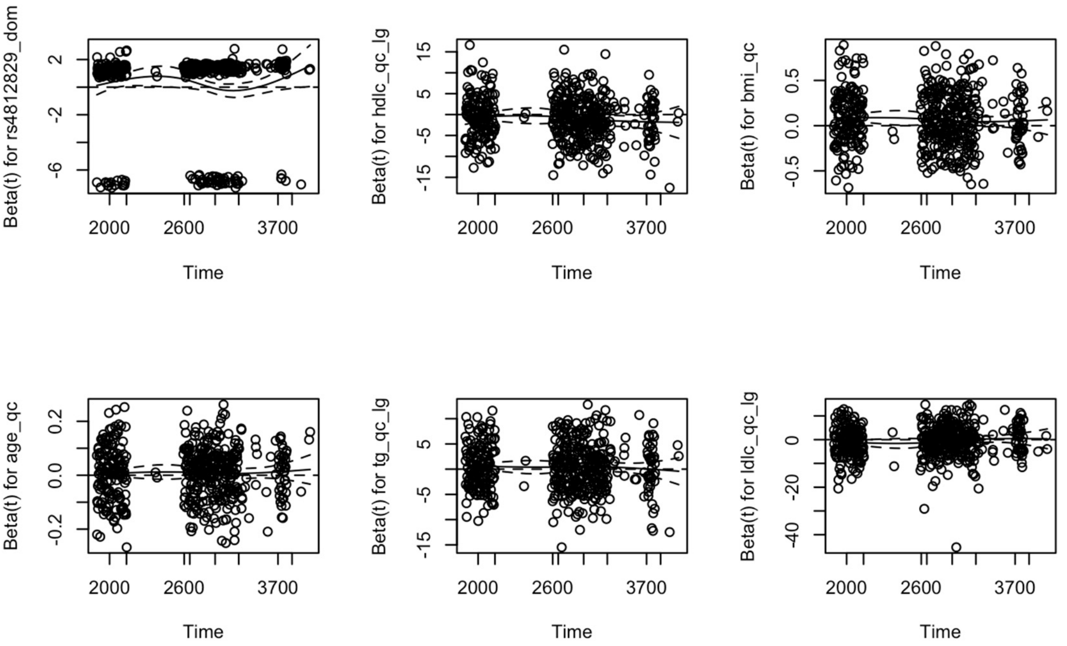

Supplementary Figure S2 Schoenfeld PH Test
